# Enhanced disinfection with hybrid hydrogen peroxide fogging in a critical care setting

**DOI:** 10.1186/s12879-022-07704-9

**Published:** 2022-09-29

**Authors:** Anjay Khandelwal, Brian Lapolla, Tina Bair, Frances Grinstead, Meaghan Hislop, Christine Greene, Michael T. Bigham

**Affiliations:** 1 Department of Surgery, Division of Burn Surgery, Paul and Carol David Foundation Burn Institute, Akron Children’s Hospital, Akron, OH USA; 2 Department of Surgery and Pediatrics, NEOMED, Rootstown, OH USA; 3 Department of Construction, Facilities and Public Safety, Akron Children’s Hospital, Akron, OH USA; 4 Department of Infection Prevention and Control, Akron Children’s Hospital, Akron, OH USA; 5 Department of Executive Management, CURIS System, 610 Kane Court, Oviedo, FL 32765 USA; 6 Department of Scientific Research, CURIS System, 610 Kane Court, Oviedo, FL 32765 USA; 7 Ramboll Group, Ramboll USA, Inc., 4245 North Fairfax Drive, Suite 700, Arlington, VA 22203 USA; 8 Department of Quality Services and Division of Pediatric Critical Care, Department of Pediatrics, Akron Children’s Hospital, Akron, OH USA; 9 Department of Pediatrics, NEOMED, Rootstown, OH USA

**Keywords:** Infection prevention, Disinfection, Hydrogen peroxide, Hospital-acquired infection, Burn, Critical care

## Abstract

**Background:**

Environmental contamination contributes to hospital associated infections, particularly those caused by multi-drug resistant organisms (MDRO). This study investigated bioburden presence on surfaces in a critical care center’s patient rooms following typical environmental services (EVS) practices and following intervention with hybrid hydrogen peroxide™ (HHP™) fogging.

**Methods:**

Upon patient discharge, following standard cleaning or cleaning with ultraviolet (UV) light use, patient rooms were sampled by swabbing for adenosine triphosphate (ATP) and aerobic colony counts (ACC) from five preset locations. Rooms were then fogged via HHP technology using chemical indicators and *Geobacillus stearothermophilus* biological indicators for sporicidal validation monitoring. Following fogging, rooms were sampled again, and results were compared.

**Results:**

A 98% reduction in ACC was observed after fogging as compared to post EVS practices both with and without UV light use. No statistical difference was seen when comparing cleaning to cleaning with UV light use. Methicillin-resistant *Staphylococcus aureus* (MRSA) and *Pseudomonas aeruginosa* were identified following EVS practices and not detected following HHP fogging. ATP samples were reduced 88% by fogging application. Chemical and biological indicators confirmed correct application of HHP fogging, as seen through its achievement of a 6-log reduction of bacterial spores.

**Conclusion:**

HHP fogging is a thorough and efficacious technology which, when applied to critical care patient rooms, significantly reduces bioburden on surfaces, indicating potential benefits for implementation as part of infection prevention measures.

## Introduction

Despite ongoing efforts to advance strategies for infection control, the challenge of preventing hospital associated infections (HAIs), particularly those caused by multi-drug resistant organisms (MDROs), persists in healthcare today. In a prevalence study conducted at 1,150 centers across 88 countries the overall rate of suspected or confirmed infection of intensive care unit (ICU) patients was 54%, higher than rates seen in previous years [1]. A ten-year study of burn injuries in pediatric patients attributed 54% of deaths to sepsis, with 73% of these caused by MDROs, of which 64% was attributed to *Pseudomonas aeruginosa* alone [2]. Patients with large burns are not only more susceptible to infection but, once infected, are more likely to shed infectious pathogens into their environment, perpetuating the contamination in burn units [3]. Once shed into the environment, organisms such as *Clostridioides difficile* (*C. diff*), methicillin-resistant *Staphylococcus aureus* (MRSA), *Acinetobacter baumannii*, and *P. aeruginosa* can survive on dry surfaces for extended time periods and are difficult to eradicate even with terminal cleaning protocols [4].

The link between environmental contamination, or bioburden, and acquired infection rates has been well studied [5–7]. Residues left by cleaning chemicals combined with organic biofilms contribute to contaminated surfaces by acting as protective reservoirs for pathogens [5, 8], with an estimated 20% of HAIs caused by transmission from environmental surfaces [9]. Two common ways healthcare facilities measure for the presence of contamination on surfaces are monitoring bioburden through adenosine triphosphate (ATP) and swabbing for aerobic colony counts (ACC) [6, 10, 11]. Although results are less rapid, ACC swabs give a more defined picture of viable bacterial presence, and high rates of these aerobic bacterial colonies have been shown to coincide with higher risk for HAIs. [10, 11]

Considering the necessity for reducing or eliminating the presence of pathogens in the patient’s environment, a novel disinfection technology, hybrid hydrogen peroxide™ (HHP™) fogging was introduced to examine its potential effects on bioburden relative to current facility practices. The HHP system delivers a hybrid mixture of vaporous and micro-aerosolized hydrogen peroxide fog via pulse technology. This novel fogging delivery accounts for naturally decomposing hydrogen peroxide by periodically replenishing the HHP fog during the disinfection cycle. This patented delivery system automatically induces the fogging dwell time, the time needed for the hydrogen peroxide fog to achieve maximum efficacy. The HHP system has demonstrated efficacy against potential HAI pathogens such as *S. aureus* and *C. difficile*. [12–15] While commonly mistaken in the healthcare environment to be equivalent in efficacy to technologies such as ultraviolet (UV) light, literature demonstrates HHP fogging and similar hydrogen peroxide (H_2_O_2_) technologies have significantly higher disinfection potential [12–19] combatting pathogens, such as *C. difficile*, norovirus, *S. aureus*, and others, including in the presence of biofilms and soil loads [13, 15, 20]. However, the impact and efficacy of HHP fogging on environmental contamination in patient rooms in a healthcare setting has not yet been measured. This work aims to examine the introduction of HHP technology in a critical care environment, quantified by ACC and ATP, and to evaluate its efficacy in countering nosocomial multi-resistant pathogens beyond currently employed practices.

## Methods

This study was undertaken in the northeast United States at a regional hospital. With over 250 annual admissions and a 6-day average length of stay, this facility’s verified adult and pediatric burn center presented a unique opportunity due to the inherent vulnerability to infection of patients recovering from burn injuries [2]. The intent of this study was to quantify potential reductions in environmental contamination through implementation of HHP fogging compared to Environmental Services (EVS) practices of standard cleaning (SC) or cleaning followed by UV light use (SC + UV), as measured by ACC and ATP. Similar to autoclaving and other sterilization procedures, HHP technology is measured with the inactivation of spore-carrying biological indicators (BIs) consistent with international standards [21]. Indicators are populated with a non-harmful colony of *Geobacillus stearothermophilus* (1 × 10^6^ and 2.2 × 10^6^ organisms) which can be disinfected alongside room surfaces giving a visible confirmation of sporicidal efficacy and providing confidence in the inactivation of lesser-resistant pathogens as well [22, 23].

Certified staff from a third-party disinfection service (ForTec Medical; Hudson, OH) conducted environmental sampling and HHP fogging implementation. Data was collected from within single occupancy patient rooms following occupancy of greater than 10 days. EVS staff performed either SC, or, for rooms where the patient had a positive MDRO culture, SC + UV (Moonbeam UVC; Diversey; Fort Mill, SC) in 2–3 locations for 3–5 min per location in accordance with manufacturer guidance [24]. Disinfection chemicals employed in these practices included: a quaternary ammonium-based product (Virex II 256; Diversey; Fort Mill, SC) marketed as a hospital grade one-step disinfectant cleaner and deodorizer (EPA 70627-24), an alcohol-based glass and surface cleaner (Glance; Diversey; Fort Mill, SC), a floor cleaner degreaser (Prominence; Diversey; Fort Mill, SC), and hydrogen peroxide wipes (Oxivir; Diversey; Fort Mill, SC) employed as needed.

**Fig. 1:**
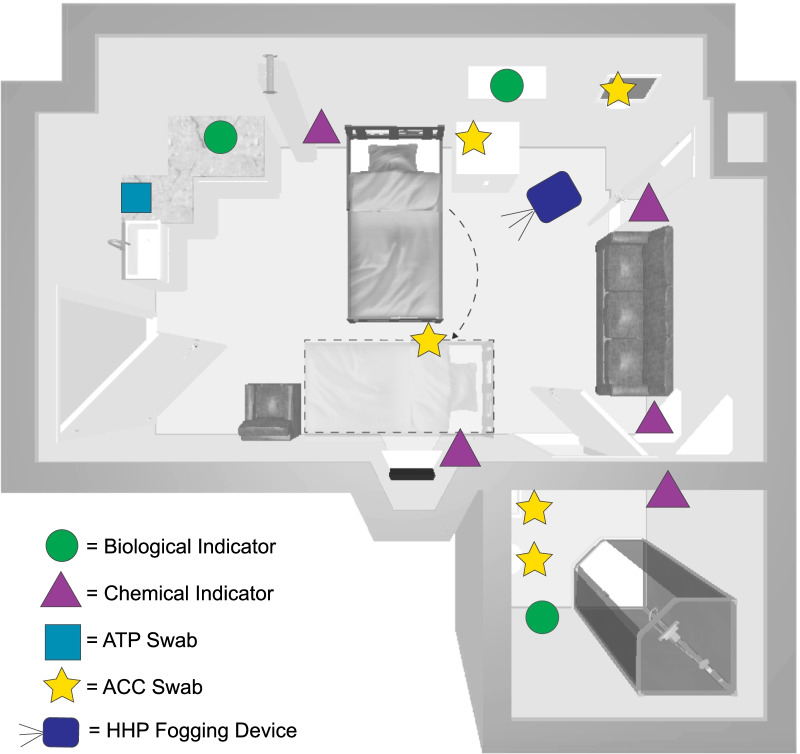
**Data Collection Locations**. This diagram of a patient room shows approximate locations where each data type was collected as well as the location of the hybrid hydrogen peroxide (HHP) fogging device. Wheeled patient beds were rotated to increase fogging access throughout the room, and returned to position following fogging. Swabbed locations ranged approximately 1 ft (0.3 m) to 10 ft (3 m) from the HHP device. Methicillin-resistant *Staphylococcus aureus* detected approximately 8 ft (2.4 m) from the HHP device was not detected following HHP fogging

Environmental sampling was performed before patient room re-occupation within 3 h of EVS practices. Swabs (Enviro swabs; 3 M; St. Paul, MN) were collected from 5 preset surfaces within the patient room: telephone, bed rail, touch screen monitor, toilet seat, and bathroom sink backsplash. Locations were selected spanning the room and adjoining bathroom in potential contact points for patients, staff, or visitors. Swabbed locations and room configuration are shown in Fig. 1. Approximately half of the rooms (7 rooms, 33–35 swabs) were sampled following SC + UV, with seven additional rooms (33–35 swabs) following SC. UV light use in the remaining three rooms was not recorded. Swabbed locations were irregular in shape, however best efforts were made to adhere to a surface area of approximately 100 cm^2^. For each swab, 25 swabbing movements were applied horizontally, vertically, and diagonally. Collected swabs were closed, sealed with paraffin tape, and maintained at 35–46°F (2–8 °C). ATP swabs (Neogen; Lansing, MI) were collected from an approximate 4 inch × 4 inch square (10 × 10 cm^2^) area of countertop adjacent to the room sink. This location was also chosen as a potential contact point, furthering the distribution of swabbed surfaces within the patient room (Fig. 1). Swabs were analyzed in the accompanying ATP Accupoint Advanced Reader (Neogen; Lansing, MI) and results recorded.

Five H_2_O_2_ chemical indicators (CIs) (3 M; St. Paul, MN) were placed throughout the room to monitor the dispersion of HHP fog. *G. stearothermophilus* BIs (1 × 10^6^ spores; Crosstex; Rush, NY) and (2.2 × 10^6^ spores; Mesa; Lakewood, CO) on steel carriers were employed as a measurement for determining adherence to the fogging protocol, failure constituting the exclusion criteria for this study. Indicators were placed throughout the room in three locations selected to challenge the HHP fog dispersion: forward of the fogging device (room sink), above and behind (patient shelf), indirect in the adjacent room (bathroom) (Fig. 1). A room could be sampled more than once if the room was reoccupied and again met the inclusion criteria.

The HHP disinfection system consists of a 7% H_2_O_2_ solution (CURoxide™ sporicidal disinfectant EPA#93324-1) paired with the CURIS portable fogging device (EPA#93324-1 CURIS System; Oviedo, FL) which together produce HHP fog. The HHP device was placed in the room along with dissipation devices (Dri-Eaz; Burlington, WA; Lasko; West Chester, PA). Dissipation devices are automatically initiated by the HHP device after the HHP cycle is complete to enhance the decomposition of any remaining HHP fog, increasing the speed at which the room is returned to initial environmental conditions. Environmental conditions were monitored to confirm the operating range ≤ 70°F (21 °C) and 25–55%Rh (Amprobe THWD-3; Everett, WA). Heating ventilation and air conditioning (HVAC) airflow was turned off, vent covers employed, doors were closed and visible gaps taped to stop air flow. Prior to the start of the study period, room dimensions for the burn center were measured from facility diagrams and dimensions were recorded in the HHP device’s data management software by the technician or authorized personnel. HHP cycle parameters were determined by room volume accounting for the drop ceiling, as calculated by the device’s software. The fogging cycle was initiated remotely from outside the room via an electronic tablet through the CURIS Decon application (app). HHP device cycle time for the rooms of this burn center averaged 42 min. Following cycle completion, the dissipation devices were initiated by the HHP device. H_2_O_2_ levels were monitored with a portable hand-held device (Dräger X-am 5100; Drager; Lübeck, DE) prior to safe re-entry.

Following dissipation, swabbing samples were collected in the same manner in immediately adjacent locations to the initial swabs. Swabs were vortexed, eluted onto tryptic soy agar plates in triplicate, incubated at 36 °C (96.8°F) for 48 h and ACC results recorded. Swabs were additionally plated on organism-specific chromogenic media (CHROMagar; Paris, FR) for identification of MRSA and *P. aeruginosa*.

CIs were collected, analyzed, and recorded. BIs were transferred into tryptic soy broth (Crosstex; Rush, NY & Mesa; Lakewood CO), incubated for 7 days at 60 °C (140°F), observed for turbidity or color change indicating growth, and results recorded. Positive BI controls accompanied each BI lot to verify viability. Challenged (exposed to HHP fog) BIs were used as a benchmark for proper sporicidal protocol and process.

Data generated from the HHP device including room parameters, HHP dosage, and cycle duration, uploaded automatically to the online data management system, and service reports were generated from that system. Information on patient length of stay and presence of MDRO (if known) was recorded. ATP results were compared post EVS practices (SC and SC + UV) and post HHP fogging. A two-sample t-test was performed to compare the non-normally distributed ATP RLU values. ACC swab results were analyzed using GraphPad Prism version 9.1.0 for Windows (GraphPad Software; San Diego, CA). The Mann–Whitney test was used to analyze post EVS and post fogging, and the Kruskal Wallis test for significance between SC, SC + UV, and post HHP fog.

## Results

During the study period of December 2020 through May 2021, 17 patient rooms were sampled. Inclusion criteria for the study was not met on three occasions when HHP fogging protocols were not followed. On these occasions data following EVS practices were unaffected and thus included in the analysis; however, no further comparison to after-HHP fogging could be made. On all other swabbing occasions, results including swabs with a non-detection were included. Patient room occupancy prior to discharge averaged 23.6 days with no detectable correlation between days occupied and baseline ACC levels (simple regression analysis, R^2^ = 0.04, *p* = 0.49). Average center occupancy was 6.5 patients, with a maximum occupancy of 12.

**Fig. 2:**
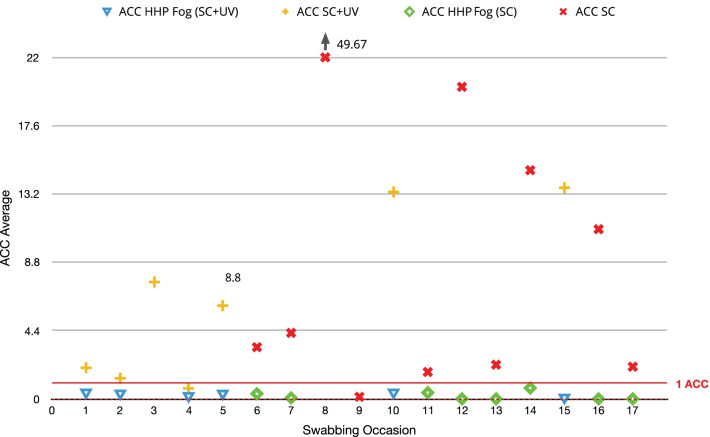
**Measurements of Disinfection Interventions**. Aerobic colony counts (ACC) following each applied intervention in relation to 1 ACC. Data show one collection below 1 ACC following standard cleaning (SC), and one collection below 1 ACC following cleaning with UV light use (SC + UV). All ACC averages following hybrid hydrogen peroxide (HHP) fogging fell below 1 ACC

A total of 150 ACC swabs were collected (82 post EVS, 68 post fogging). Following EVS practices (SC and SC + UV), 5 swabbed surfaces ranged from 0 to 153 ACC (mean 8.357 ACC, SD = 21; median 1.17 ACC). 63.4% of samples detected bacterial presence, with 85% of these demonstrating a result greater than or equal to 1 ACC. Following HHP fogging, ACC ranged from 0 to 1.33 ACC (mean 0.137 ACC, SD = 0.295; median 0 ACC) for a further reduction of 98% compared to post EVS practices (Mann Whitney test *p* < 0.0001) (Fig. 2). 23.5% of samples detected bacterial presence with only one sampled location averaging greater than 1 ACC (1.3 ACC). To evaluate the impact of UV light use (SC + UV) relative to standard cleaning (SC), HHP fogging, or any cumulative effect (SC + UV followed by HHP fog), data from these groups was compared using a Kruskal Wallis, uncorrected Dunn’s test. Application of HHP fog was statistically different from both SC and SC + UV (*p* = 0.0002 and *p* < 0.0001, respectively). However, there was no statistical difference between standard cleaning (SC) and UV light use (SC + UV) (*p* = 0.186).

MRSA was detected on five sampling occasions, following both SC and SC + UV (mean 0.1788 ACC, SD = 1.298). Four of the five occasions with MRSA results met the inclusion criteria and demonstrated a 100% reduction of MRSA (Table 1). Of the 82 total samples collected, samples positive for MRSA represented 6.1%. *P. aeruginosa* was detected twice following EVS practices (mean 0.1545, SD = 1.399). This small sample size did not allow for a full statistical analysis, however the swabbing occasion with the maximum value of 12.67 colonies was reduced 100% following fogging. *P. aeruginosa* represented 2.44% of the overall collected samples.

**Table 1:** **Methicillin-resistant** ***Staphylococcus aureus* (MRSA) data**. Four swabbing occasions allowed for a comparison between MRSA detected following environmental services (EVS) standard cleaning (SC) or cleaning with ultraviolet (UV) light use (SC + UV), and following HHP fogging. MRSA detected post SC or SC + UV was not detected following HHP fogging indicating a 100% reduction of MRSA

Swabbing occasion	#5	#6	#12	#16
Post EVS MRSA colony counts	0,0,1	0,0,1	0,0,3	8,10,17
Post EVS MRSA average	0.33	0.33	1.0	11.67
Post HHP fogging MRSA	**0**	**0**	**0**	**0**

Following EVS practices (SC and SC + UV), mean ATP results were 9,021 (SD = 9,780) relative light units (RLU). Following HHP fogging, ATP averaged 1,109 RLU (SD = 855) for an overall average reduction of 88%. A two-sampled t-test revealed a statistically significant difference in mean RLU after EVS practices and RLU following HHP fogging (*p* = 0.013).

Beginning with swabbing occasion 12, the number of BIs was reduced as there was a national shortage of available indicators due to the need to validate disinfection processes during the COVID-19 pandemic. For rooms including two BIs, the forward location adjacent to the room sink was excluded as the other two locations were thought to represent a greater challenge to inactivation of the BIs. Sample occasions 14–17 employed one BI as previously described and one indicator with a *G. stearothermophilus* population of 2.2 × 10^6^, where the higher population indicator was placed on the shelf. There were three instances of BI failures coinciding with a failure of fogging protocol. No other failed indicators were observed, for a total of 37 of 43 indicators demonstrating a greater than 6-log sterilization of challenged indicators. 85 CIs were collected over the course of study. CIs consistently demonstrated hydrogen peroxide exposure in the farthest reaches of each room, even in rooms where BI failures occurred.

## Discussion

To meet the need for disinfecting environmental surfaces, an ever-increasing amount of disinfection technologies are available, however, not all types of advanced disinfection tools are similar in their outcome. While these differences in efficacy are demonstrated across a bulk of research [15, 18, 19], the damaging perception remains, leading to ineffective disinfection practices despite the cost and effort of implementation. The range of present pathogens is approached in a uniform manner, which ignores differences in their susceptibility to disinfectants [5, 23, 25], and important pathogenic behavior such as the potential for environmental persistence and bacterial regrowth [4, 5, 7, 8, 26]. Given the increased tolerance to disinfection of bacteria within biofilms over isolated bacterium [5, 8, 27], as well as the ability of organisms in biofilms to confer antibiotic resistance [5, 8, 27, 28], this oversight could have serious consequences such as rapid regrowth to an infectious dose [26, 29] or development of disinfectant resistance [27]. Even non-chemical disinfection technologies such as UV light can result in a lesser log reduction of pathogens [18, 19, 30]. In light of the severity of outcome and resulting high mortality rates caused by infection with an MDRO [2, 3], infection control practices intended to remove the risks posed by contaminated environmental surfaces must reach beyond a partial disinfection and target a complete eradication of environmental pathogens.

These results were evaluated to compare the effects of HHP fogging to current facility practices, demonstrating HHP significantly reduced environmental contamination. Following HHP fogging, ACC in patient rooms dropped 98% from levels post SC and SC + UV, consistently averaging less than one colony count. All locations where MRSA was identified post EVS practices were negative for MRSA following HHP fogging. These results in combination with laboratory testing [12, 13] showing efficacy against *S. aureus* possibly indicate this process is effective in combating MRSA, however due to the small sample size, further investigation is merited. Likewise, on the swabbing occasion with the greatest detected colony count, *P. aeruginosa* was reduced from 12.6 ACC following EVS practices to 0 ACC following HHP fogging. This study was not powered to evaluate HHP fogging’s impact on HAIs, though the reduction of identified bacterial environmental contamination (a known factor in HAIs) is promising.

The ACC data collected following SC and SC + UV reflected a wide variance in bacterial levels, with 63.4% of tested surfaces remaining contaminated. This finding is neither unexpected nor surprising, as it is well understood that manually applied cleaning methods are inherently imperfect despite best application efforts [11]. The further comparison of SC + UV rooms versus SC rooms showed no statistically significant difference in ACC levels, which may be in accordance with known challenges affiliated with UV light use, such as shadows, run time, and distance [19, 30]. Further, MRSA was detected on five occasions and *P. aeruginosa* on two occasions, irrespective of UV light application, affirming the known difficulties in eliminating these common pathogens. MDRO’s survivability as well as rapid propagation create particular risk of infection for the subsequent room occupant, emphasizing the importance of achieving their elimination [11].

Biological indicators introduced into the room to monitor the HHP fogging cycle repeatedly resulted in successful inactivation demonstrating sporicidal efficacy of the disinfection treatment and indicating efficacy against less hardy pathogens [16, 22, 23]. The HHP system has been effective against *C. difficile* spores in a three-part soil load [14, 15], which, in combination with the sporicidal efficacy seen in this study, suggests that it may likewise be used for combatting the fastidious and pathogenic *C. difficile* in a healthcare environment. On three occasions, a less than 6-log reduction of one or more indicators coincided with failures in adherence to fogging protocols which require airflow control within the room for the duration of the fogging cycle. Fogging technologies must be contained within the room to achieve optimal contact of the disinfectant. Incomplete vent coverage as well as activation of the facility’s HVAC before HHP cycle completion did not allow for optimal contact. These results demonstrate the transparency of biological efficacy in real time and confirm the applicability of BIs as an effective tool for monitoring the proper use of this technology.

Despite rigid cleaning protocols and heightened awareness of infection risks in a critical care environment, including burn units, ATP levels post EVS practices were consistently found to be high [31]. While ATP is a practical measurement tool, RLU indicates a measure of organic presence, but it is unable to distinguish between pathogenic organisms and other organic materials and can be influenced by cleaning chemicals and their residues [11, 32]. High ATP readings may therefore be a combination of chemical and biological factors, possibly indicating the presence of biofilms and residues which can promote pathogen reservoirs [5, 8, 11, 27]. Post HHP fogging, ATP RLUs were reduced in every room, which is particularly notable as no wiping of the surface took place between swabbing. The observed reduction therefore could be due to biological breakdown, chemical residues, or a combination of these factors. Though lacking in specificity, measurements of ATP are commonly used as a monitoring tool for environmental cleanliness in healthcare facilities, due in part to the ability to obtain rapid results. ATP swabbing was included in this study design for its relevance to other facilities and existing literature [31].

The reduction in ACC, and ATP RLU’s are strong indications that bioburden present after EVS practices have been inactivated or reduced by HHP fogging to levels associated with a risk reduction for HAIs from environmental surfaces [11, 31, 33]. In addition to being effective against difficult to kill pathogens [15], HHP fogging presents an inherent advantage in terms of whole room dispersal [17] and reaching areas where wiping alone or UV light may not adequately reach [30]. The results presented emphasize HHP fogging achieves more efficacious disinfection of room surfaces than manual cleaning and UV light application combined.

There are some limitations to this study. Though typical patient volumes were seen, the time period studied overlapped with enhanced cleaning practices in response to the COVID-19 pandemic, limiting the ability to compare these results with those of a similar time period. In consideration of the enhanced demands placed on the environmental services team during the pandemic, the EVS staff was not blinded for the purposes of this study. This awareness may have influenced the individual practices of staff members. This study focused on efficacy of outcome as the most important consideration for disinfection practices. Due to the additional time needed to collect samples and place indicators to determine efficacy, the time needed for each disinfection intervention was not recorded, though may be of interest. The facility’s application of UV light following known MDRO cases enabled comparisons presented here; however, additional head-to-head investigations would be of interest to aid in the understanding that disinfection technologies have wide ranges in achievable efficacy, particularly in real-world applications. Though detected, the small sample size of MRSA and *P. aeruginosa* limited the ability to draw statistical inferences despite the data that was obtained. Further study regarding disinfection of these and other epidemiologically important pathogens in the healthcare setting is needed. Although this testing targeted bacterial counts as well as specific pathogens, a further investigation into the impact of HAIs within the tested department is warranted.

In closing, this study examined patient rooms of a critical care center, finding that HHP fogging following current EVS practices, with or without UV light use, resulted in a significant reduction in bacterial presence on the environmental surfaces. The confirmed sporicidal efficacy may denote significantly lower risk posed by environmental contamination and play an important role in preventing HAIs. This real-world application of HHP fogging demonstrates a thorough and efficacious treatment method for patient spaces, indicating integration of HHP technology in hospitals could further enhance infection prevention.

## Data Availability

The datasets used and/or analyzed during the current study are available from the corresponding author on reasonable request.

## Abbreviations

MDRO: Multi-drug resistant organisms
EVS: Environmental services
SC: Standard cleaning
SC + UV: Standard cleaning with UV light use
HHP: Hybrid hydrogen peroxide
ATP: Adenosine triphosphate
RLU: Relative light units
ACC: Aerobic colony counts
UV: Ultraviolet
HAIs: Hospital associated infections
MRSA: Methicillin-resistant *S**taphylococcus aureus*
